# Associations of self-efficacy and outcome expectancy with adherence to continuous positive airway pressure therapy in Japanese patients with obstructive sleep apnea

**DOI:** 10.20407/fmj.2022-015

**Published:** 2022-10-28

**Authors:** Shigeko Kojima, Ayako Saito, Fumihiko Sasaki, Masamichi Hayashi, Yuki Mieno, Hiroki Sakakibara, Shuji Hashimoto

**Affiliations:** 1 Department of Rehabilitation, Faculty of Health Sciences, Nihon Fukushi University, Handa, Aichi, Japan; 2 Department of Hygiene, Fujita Heath University, School of Medicine, Toyoake, Aichi, Japan; 3 SDB Research Laboratory, Takaoka Clinic, Nagoya, Aichi, Japan; 4 Division of Respiratory Medicine, Fujita Health University Okazaki Medical Center, Okazaki, Aichi, Japan; 5 Department of Respiratory Medicine, Fujita Health University, School of Medicine, Toyoake, Aichi, Japan; 6 Tokushige Kokyuki Clinic, Nagoya, Aichi, Japan

**Keywords:** Obstructive sleep apnea, Continuous positive airway pressure, Self-efficacy, Outcome expectancy, Adherence

## Abstract

**Objective:**

To examine the associations of self-efficacy and outcome expectancy with adherence to continuous positive airway pressure (CPAP) therapy among Japanese men with obstructive sleep apnea (OSA) using objective adherence data for CPAP therapy.

**Methods:**

We conducted a retrospective study of 497 Japanese men with OSA who were receiving CPAP therapy. Good adherence was defined as CPAP use of ≥4 hours per night for ≥70% of nights. Logistic regression models were used to calculate odds ratios (ORs) and 95% confidence intervals (CIs) for the associations of good adherence to CPAP therapy with self-efficacy and outcome expectancy (measured with the CPAP Self-Efficacy Questionnaire for Sleep Apnea in Japanese). The models were adjusted for age, duration of CPAP therapy, body mass index, apnea–hypopnea index, Epworth Sleepiness Scale score, and comorbidities (diabetes mellitus and hypertension).

**Results:**

In total, 53.5% of participants had good adherence to CPAP therapy. The mean CPAP use was 5.18±1.53 hours/night. After adjusting for related factors, we found significant associations of good adherence to CPAP therapy with self-efficacy scores (OR, 1.10; 95% CI, 1.05–1.13; *p*<0.001) and outcome expectancy scores (OR, 1.10; 95% CI, 1.02–1.15; *p*=0.007).

**Conclusions:**

Our results indicate that self-efficacy and outcome expectancy are associated with good adherence to CPAP therapy among Japanese men with OSA.

## Introduction

Obstructive sleep apnea (OSA) is a common clinical condition characterized by repetitive upper airway collapse during sleep; it is a known risk factor for metabolic disease, hypertension, neurological disorders, cardiovascular disease, and mortality.^1–6^ Globally, an estimated 425 million people have moderate to severe OSA, for which treatment is generally recommended.^7^

Continuous positive airway pressure (CPAP) is considered the first-line therapy for OSA. CPAP reduces snoring, hypoxemia, and resultant arousals in patients with OSA, thereby improving their quality of life and neurocognitive function. Patients’ use of CPAP is critically important for effective treatment of OSA. However, adherence to CPAP therapy has been objectively measured from the records of CPAP devices, revealing low adherence.^8^ Low adherence to CPAP therapy among patients with OSA has been associated with several factors, including sociodemographic characteristics, disease severity, side effects, and psychosocial factors.^8–10^

Bandura’s social cognitive theory posits that there are multiple influences on behavior, with an emphasis on personal (e.g., cognitive) and environmental (e.g., social) factors.^11,12^ Psychosocial factors derived from this model include self-efficacy and outcome expectation. For patients with OSA treated with CPAP therapy, self-efficacy refers to the confidence and ability to self-manage CPAP treatment, and outcome expectancy refers to the self-perceived expectation of benefiting from CPAP treatment. Many studies have examined associations of adherence to CPAP therapy with self-efficacy and outcome expectancy in patients with OSA treated with CPAP therapy, but their results were inconsistent.^12–20^ In Asian patients with OSA, including Japanese patients, no studies have explored the association of self-efficacy and outcome expectancy with adherence to CPAP therapy using objective adherence data.

In the present study, we examined associations of adherence to CPAP therapy with self-efficacy and outcome expectancy among Japanese men with OSA using adherence data recorded with a CPAP device.

## Methods

### Participants

This study involved 998 Japanese men with OSA who were being treated with CPAP at SDB Research Laboratory, Takaoka Clinic, in August 2011. The patients’ apnea–hypopnea index (AHI) before CPAP therapy was obtained from overnight polysomnography recordings (Embla N7000; Embla Systems, Inc., Broomfield, CO, USA). The data obtained from these recordings included a continuous electroencephalogram, oculogram, electrocardiogram, electromyogram, respiratory channels including nasal and oronasal airflow, thoracic and abdominal respiratory movements and pulse oximetry, snoring, position, and video monitoring. Sleep stages and respiratory events were scored using the standard diagnostic criteria of the American Academy of Sleep Medicine and a criterion published by registered polysomnogram technicians.^1^ All participants in this study were patients with OSA who had an AHI of ≥20 events/hour or 5 to 19 events/hour and had undergone failed therapy with an oral appliance.^21^ Participants were excluded if therapy with their oral appliance was successful.

### Survey procedure

Of the 998 participants, 705 (71%) completed a self-administered questionnaire in August 2011. The questionnaire included the CPAP Self-Efficacy Questionnaire for Sleep Apnea in Japanese (CSESA-J),^22^ the Epworth Sleepiness Scale (ESS),^23^ and items regarding the patients’ medical history. Weight was also self-reported in the questionnaire. The patients’ AHI before CPAP therapy, age, and height were obtained from clinical records at SDB Research Laboratory, Takaoka Clinic. Their body mass index (BMI) was calculated as weight divided by height in meters squared (kg/m^2^). Of 705 survey respondents, 579 (82%) provided their CPAP device with a smart card system, which records data on adherence to CPAP therapy.

The mean±standard deviation (SD) age and AHI of the 705 survey respondents was 56.0±11.2 years and 49.0±26.0 events/hour, respectively. The mean±SD age and AHI of the 579 patients who provided CPAP devices were 56.0±11.1 years and 49.8±26.0 events/hour, respectively, which were not significantly different from those for all survey respondents.

### CPAP adherence

We obtained data on the participants’ CPAP therapy adherence, which was objectively measured by a smart card system. Using the data for 1 month after the patient completed the questionnaire, we classified CPAP use of ≥4 hours per night for ≥70% of nights as good adherence and other CPAP use as poor adherence.^24^

### Self-efficacy measurement

We used the CSESA-J to measure participants’ self-efficacy and outcome expectancy. The CSESA-J is a scale for Japanese patients with OSA treated with CPAP under the Bandura’s social cognitive theory and has two subscales: self-efficacy and outcome expectancy.^22^ Its reliability and validity have been confirmed.

### Data analyses

Of the 579 patients, we excluded 13 patients with missing questionnaire data, 35 female patients, and 36 patients with a history of cerebral or cardiovascular disease. Therefore, 497 patients with valid data were included in the analyses.

The average age, BMI, and AHI were compared between the good and poor adherence groups using independent-samples *t*-tests. The median and interquartile range were used to examine the duration of CPAP therapy and ESS score, and these factors were analyzed with the Wilcoxon rank sum test because of the large distortion in the data distribution. Logistic regression models were used to calculate the odds ratios (ORs) and 95% confidence intervals (CIs) for the associations of good adherence to CPAP therapy with self-efficacy and outcome expectancy scores, adjusted for age, duration of CPAP therapy, BMI, AHI, ESS score, and comorbidities (diabetes mellitus and hypertension).^9,10,25^ The level of statistical significance was set at 0.05. The analyses were performed using IBM SPSS Version 23 for Windows (IBM Japan Ltd., Tokyo, Japan).

### Ethical considerations

All participants provided written informed consent before completing the self-administered questionnaire. This study was approved by the Fujita Health University Medical Research Ethics Committee, Aichi, Japan in November 2010 (10-198) and was conducted in accordance with the Declaration of Helsinki.

## Results

**Figure 1:**
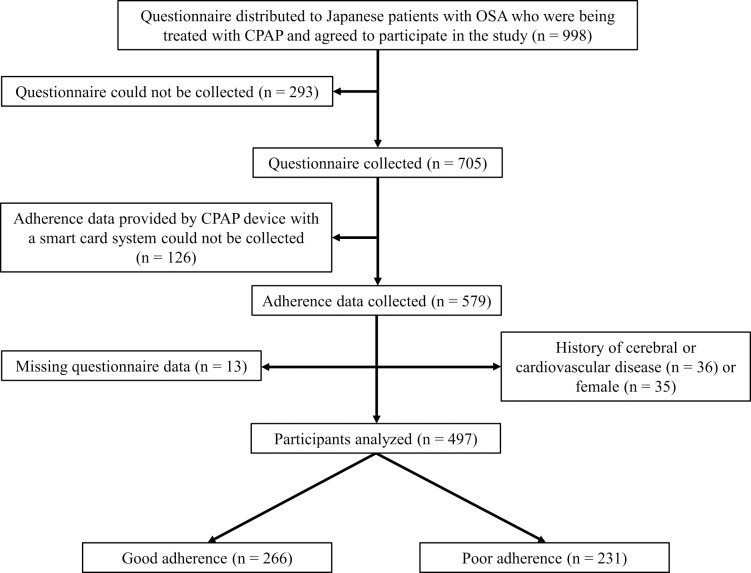
Flow diagram of study participants Abbreviations: CPAP, continuous positive airway pressure; OSA, obstructive sleep apnea

Figure 1 shows the flow diagram of the study participants. Of the 497 participants analyzed, 266 (53.5%) had good adherence and 231 (46.5%) had poor adherence. The mean±SD duration of CPAP use was 5.18±1.53 hours/night. The participants’ characteristics and CSESA-J scores stratified by adherence group are shown in Table 1. There were no significant differences in mean age, mean BMI, mean AHI, median duration of CPAP therapy, median ESS score, or percentage of comorbid hypertension between the poor and good adherence groups. The percentage of patients with comorbid diabetes mellitus was significantly lower in the good adherence group than in the poor adherence group (*p*=0.024). The mean±SD self-efficacy and outcome expectancy scores in the good adherence group were 35.9±6.1 and 22.1±3.9, respectively, and those in the poor adherence group were 32.2±6.1 and 20.6±3.9, respectively. There were significant differences in the mean self-efficacy and outcome expectancy scores between the two adherence groups (Table 1).

The ORs and 95% CIs for the associations of good adherence to CPAP therapy with self-efficacy and outcome expectancy scores are shown in Table 2. After adjusting for age, duration of CPAP treatment, BMI, AHI, ESS score, and comorbidities (diabetes mellitus and hypertension), there were significant associations of good adherence to CPAP therapy with self-efficacy scores (OR, 1.10; 95% CI, 1.05–1.13; *p*<0.001) and outcome expectancy scores (OR, 1.10; 95% CI, 1.02–1.15; *p*=0.007) (Table 2).

## Discussion

This is the first study to focus on the associations of self-efficacy and outcome expectancy with adherence to CPAP therapy based on objective adherence data among Asian patients with OSA. We found that self-efficacy was significantly associated with good adherence to CPAP therapy among Japanese men with OSA. Our finding is consistent with the results of most previous studies.^13,15–20^ These previous studies targeted different populations in Australia,^13^ the United States (Whites, Blacks, and Hispanics),^15,16,19,20^ Finland,^18^ and China.^17^ These studies used two scales to measure self-efficacy and outcome expectancy: the Social Cognitive Theory (SCT) scale and the Self-Efficacy Measure for Sleep Apnea questionnaire (SEMSA).^26,27^ These two scales were designed for Western patients. The CSESA-J^22^ used in the present study was developed for Japanese patients. All three scales are based on Bandura’s social cognitive theory. The present study confirmed the positive association between self-efficacy and good adherence to CPAP therapy using the CSESA-J among Japanese patients.

Our study also indicated that outcome expectancy was significantly associated with good adherence to CPAP therapy. Two previous studies conducted in China^17^ and Australia^14^ showed a positive association similar to that in our study, whereas three studies conducted in Australia^13^ and the United States^15,16^ showed no such association. Outcome expectancy may only be a cognitive factor in patients with OSA who have used CPAP treatment for a relatively long time because it reflects the self-perceived expectation of benefiting from CPAP treatment. Although there were many differences between those studies, including the present study, the mean duration of CPAP use per night in the two previous studies^14,17^ and the present study showing a positive association was 4.6 to 5.3 hours/night, which tended to be longer than in the three studies showing no association^13,15,16^ (2.5–3.2 hours/night). This suggests that there may be a positive association between outcome expectancy and adherence to CPAP therapy in a population with a relatively high adherence rate to CPAP therapy. If true, this finding will be helpful to determine the content of education that is needed for patients to improve their adherence to CPAP therapy. When directed toward altering patients’ views about CPAP, behavioral interventions such as cognitive behavioral therapy and motivational enhancement therapy appeared to be effective in promoting adherence.^12^ Further research is needed to clarify the relationship between adherence to CPAP therapy and outcome expectancy in patients with OSA.

This study had several limitations. First, no unified definition of adherence is available, and previous studies have shown that the prevalence of good adherence among people being treated with CPAP ranges from 30% to 60%.^8^ We used a common definition of good adherence as the use of CPAP for ≥4 hours per night for ≥70% of nights.^24^ Second, our study was retrospective in nature and conducted at a single clinic for sleep-disordered breathing. Third, our participants were patients who continued medical treatment for OSA and did not include those who discontinued treatment. We were not able to examine the status of CPAP treatment discontinuation. Two previous studies from the United States^16,19^ also included patients with OSA who were receiving CPAP therapy and were already being followed up. Fourth, the collection rate in this study was not high, but the mean age and AHI were similar between the 705 participants who completed the questionnaire and the 579 participants with valid data on CPAP therapy adherence. Fifth, all participants in this study were male because there were few female patients at the studied clinic. A previous study showed that sex may be associated with CPAP adherence.^9^ Although the percentage of female patients with OSA is not high, a further study with data obtained from more female subjects would provide important information. The final limitation is that the adjustment factors were selected based on previous studies; these factors included age, duration of CPAP therapy, BMI, AHI, ESS score, and comorbidities (diabetes mellitus and hypertension).^9,10,25^ It could have been helpful to include other factors that might have influenced our results (e.g., insomnia, subjective symptoms, socioeconomic status, and education level).^9^ Although it would have been useful to include these factors in the analyses, this information was not available in our study.

In conclusion, our results indicate that self-efficacy and outcome expectancy are significantly associated with good adherence to CPAP therapy in Japanese men with OSA.

## Figures and Tables

**Table1:** Characteristics and CSESA-J scores of Japanese men with OSA receiving CPAP therapy

	Good adherence (*n*=266)	Poor adherence (*n*=231)	*p*-value
Characteristics			
Age, years	56.2±11.6	54.4±10.2	0.081
Duration of CPAP therapy, years	1.2 (0.5–5.9)	1.5 (0.6–5.1)	0.871
BMI, kg/m^2^	28.4±4.9	28.4±5.5	0.898
AHI, events/hour	56.2±22.8	53.6±21.6	0.220
ESS score	8.0 (4.0–11.0)	9.0 (6.0–13.0)	0.110
Comorbidities			
Diabetes mellitus	55 (44.0)	70 (56.0)	0.024
Hypertension	57 (55.3)	46 (44.7)	0.908
CSESA-J			
Self-efficacy score	35.9±6.1	32.2±6.1	<0.001
Outcome expectancy score	22.1±3.9	20.6±3.9	<0.001

Data are presented as mean±standard deviation, median (interquartile range), or n (%). Abbreviations: AHI, apnea–hypopnea index; BMI, body mass index; CPAP, continuous positive airway pressure; CSESA-J, CPAP Self-Efficacy Questionnaire for Sleep Apnea in Japanese; ESS, Epworth Sleepiness Scale; OSA, obstructive sleep apnea.

**Table2:** Associations of good adherence to CPAP therapy with self-efficacy and outcome expectancy in Japanese men with OSA

	OR (95% CI)	*p*-value	Adjusted OR (95% CI)	*p*-value
CSESA-J				
Self-efficacy	1.10 (1.07–1.14)	<0.001	1.10 (1.05–1.13)	<0.001
Outcome expectancy	1.10 (1.05–1.16)	<0.001	1.10 (1.02–1.15)	0.007

Abbreviations: CI, confidence interval; CPAP, continuous positive airway pressure; CSESA-J, CPAP Self-Efficacy Questionnaire for Sleep Apnea in Japanese; OR, odds ratio; OSA, obstructive sleep apnea. Logistic regression models were used to calculate the OR and 95% CI for the associations of good adherence to CPAP therapy with self-efficacy and outcome expectancy scores, adjusted for age, duration of CPAP therapy, body mass index, apnea–hypopnea index, Epworth Sleepiness Scale score, and comorbidities (diabetes mellitus and hypertension).
